# Révision de la sous-famille des Metaracoelophryinae de Puytorac 1972 (Oligohymenophora : Hoplytophryida : Hoplytophryidae), ciliés astomes du tube digestif d’oligochètes terricoles d’Afrique : description de cinq espèces nouvelles

**DOI:** 10.1051/parasite/2012191041

**Published:** 2012-02-15

**Authors:** Z. Fokam, P. Ngassam, P.A. Nana, G. Bricheux, P. Bouchard, T. Sime Ngando

**Affiliations:** 1 Laboratoire de Biologie Générale, Faculté des Sciences, Université de Yaoundé I BP 812 Yaoundé Cameroun; 2 Department of Biology, Higher Teacher Training College, University of Bamenda, Cameroon P.O. Box 39 Bamenda Cameroun; 3 Clermont Université, Université Blaise Pascal, LMGE BP 10448 63000 Clermont-Ferrand France; 4 CNRS, UMR6023 BP 80026 63171 Aubière Cedex France

**Keywords:** *Paracoelophrya*, *Dicoelophrya*, *Alma*, imprégnation argentique, DAPI, cilié, astome, *Paracoelophrya*, *Dicoelophrya*, *Alma*, silver staining, DAPI, astome, ciliate

## Abstract

Cinq nouvelles espèces de ciliés astomes, présentes dans le tube digestif de vers oligochètes du genre *Alma* du Cameroun, ont été décrites. Les techniques utilisées sont : l’observation vitale, la coloration au DAPI, la microscopie électronique à balayage et les imprégnations argentiques selon Fernandez Galiano, 1966. Ce travail confirme la présence des genres *Paracoelophrya* et *Dicoelophrya* dans le tube digestif des oligochètes du genre *Alma* du Gabon et du Cameroun ; il permet de faire une synthèse récapitulative de la sous-famille des Metaracoelophryinae. De plus, est confirmée l’homogénéité de ce groupe, et est reposée la question de la parenté phylogénétique des Hoplitophryida.

## Abstract (en)

Five new species belonging to the astome ciliates, living in the digestive tract of Oligochaeta worms belonging to the genus *Alma* from Cameroon, have been described. The techniques used are: vital staining, staining of the nucleus with Diamidino Phenyl Indol (DAPI), scanning electron microscopy and silver staining method (Fernandez Galiano, 1976, 1994). This work confirms the presence of the genus *Paracoelophrya* and *Dicoelophrya* in the digestive track of the oligochaete *Alma* from Gabon and Cameroon; it helps to understand the general taxonomy of this Metaracoelophryinae subfamily. Moreover, the homogeneity of this group is confirmed and the phylogenetic relationship inside the Hoplitophryida order need more studies to be solved.

## Introduction

Dans la classe des Oligohymenophora de Puytorac (1974), les ciliés de la sous-classe Astomatia Schewiakoff, 1896 sont caractérisés essentiellement par l’absence d’une cavité buccale. De ce fait, les critères généralement utilisables pour la systématique se focalisent sur les caractéristiques du cinétome et sur celles d’un appareil squelettique lorsqu’il est présent. La diversité des astomes est remarquable en raison de leur présence dans le tube digestif des oligochètes essentiellement, mais aussi de quelques polychètes, triclades, mollusques et batraciens. De nombreuses données ont été acquises (de Puytorac & Schrével, 1965; de Puytorac & Dragesco, 1968, 1969; Ngassam, 1983; de Puytorac, 1994; Fokam *et al.*, 2008).

Les Hoplitophryida sont porteurs d’un appareil squelettique au moins présent sur la partie antérieure de la cellule et aboutissant à une pièce en V. Dans cet ordre sont incluses les familles des Hoplitophryidae Cheissin, 1930 emend de Puytorac, 1972, Radiophryidae de Puytorac, 1972, Contophryidae de Puytorac, 1972, Maupasellidae Cépède, 1910 et Intoshellinidae Cépède, 1910.

Dans les Radiophryidae, aux sous-familles Eudrilophryinae de Puytorac, 1971, Radiophryinae Rossolimo, 1929 emend de Puytorac, 1972, Anthonyellinae de Puytorac, 1972, Metaradiophryinae de Puytorac, 1972, Durchoniellinae de Puytorac, 1972, Acanthodiophryinae de Puytorac, 1972, a été ajoutée la sousfamille Metaracoelophryinae par de Puytorac, 1972. Cette sous-famille Metaracoelophryinae est caractérisée par un cytosquelette antérieur, formé d’un élément en V porteur d’un crochet plus ou moins marqué et, sur la face inférieure de la cellule, de fibres squelettiques. Ces dernières s’épaississent sur une partie de leur parcours, en arceaux qui renforcent une gouttière plus ou moins étendue au milieu ou de part et d’autre de la cellule. La ligne antérieure de suture des cinéties se prolonge en deux systèmes sécants. Une aire thigmotactique antérieure peut être présente. Il y a deux à plusieurs systèmes sécants postérieurs.

Quatre genres ont été décrits : *Metaracoelophrya* de Puytorac & Dragesco, 1968; *Paracoeloprya* de Puytorac 1969; *Coelophrya* de Puytorac & Dragesco, 1969; *Dicoelophrya* de Puytorac & Dragesco, 1969. Nous avons repris l’étude des représentants de cette sous-famille en examinant le tube digestif des vers Glossoscolecidae du genre *Alma; A. emini* et *A. nilotica*, provenant de plusieurs localités du Cameroun.

## Matériel et Méthodes

**Figure 1.**
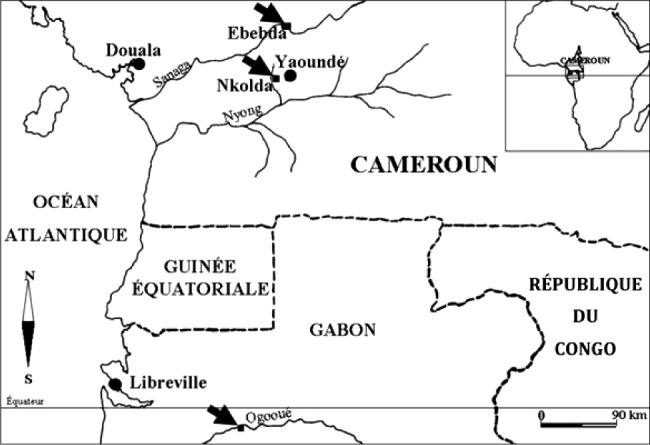
Carte de la région d’Afrique Centrale montrant les trois localités où sont connues des populations de Metracoelophyinae. Les flèches indiquent les lieux précis des stations.

Les vers étudiés ont été récoltés dans trois stations du Cameroun : Ebebda (berges de la Sanaga), Nkolda et Nkolbikogo, deux villages voisins, sur les berges de la Mefou, affluent du Nyong (Figure 1). La récolte des vers a été réalisée par des saignées profondes et rapides dans le sol, à proximité des tortillas. Les vers ont été ensuite identifiés selon les clés d’Omodeo, 1958 et conservés dans la terre humide pendant une semaine maximum pour éviter la défaunation. Des fragments du tube digestif sont dilacérés dans du liquide physiologique de Ringer ou de l’eau minérale du commerce Supermont (HCO3 128,0 mg/L, Cl^-^ 2,1mg/L, SO4 - 1,0 mg/L, NO3 - 4,1 mg/L, Ca^++^ 16,0 mg/L, Mg^++^ 10,2 mg/L, K^+^ 5,2 mg/L et Na^+^ 12,7 mg/L). Les ciliés, qui nagent entre les fragments du tube digestif, sont triés à la micropipette sous la loupe binoculaire Wild M5. L’étude de la ciliature somatique et des structures fibrillaires est effectuée à la lumière d’imprégnations argentiques suivant la méthode de Fernandez Galiano (1976, 1994). L’appareil nucléaire a été coloré à l’aide de la réaction de Feulgen (Blanc, 1965) ou par coloration au 4’,6-diamidino-2-phenylindole (DAPI) (Williamson & Fennell, 1975) pour une observation en microscopie à fluorescence. Les mesures sont obtenues à partir de 30 cellules de chaque espèce. Les dessins sont réalisés à la chambre claire du microscope Wild M5. Pour la microscopie à balayage, les cellules isolées à la pipette sont fixées au glutaraldehyde 4% puis à l’OsO4 1% (dans du tampon cacodylate 0,1 M). Après déshydratation, les ciliés sont observés sur un microscope Jeol 6060.

## Résultats

### Description des espèces du genre *Paracoelophrya*

Nous présentons trois nouvelles espèces du genre *Paracoelophrya* : *P. falcifera*, *P. polymorphus* et *P. ebebdensis*.

### *Paracoelophrya falcifera* n. sp.

**Figure 2.**
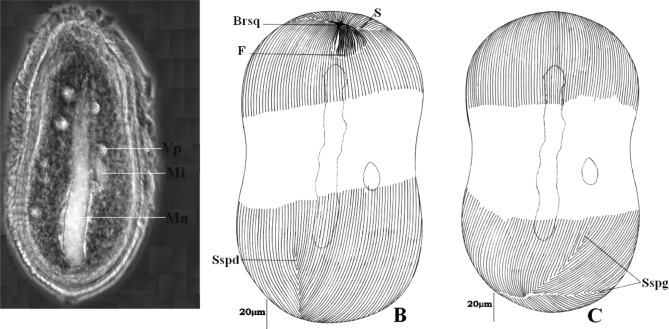
*Paracoelophrya falcifera* n. sp. A : morphologie générale; image de microscopie à contraste de phase, superposée à la fluorescence de l’appareil nucléaire coloré au DAPI; B : ciliature de la face inférieure; C : ciliature de la face supérieure. Mn : macronoyau; Mi : micronoyau; Vp : vacuole pulsatile; F : fibres squelettiques; Sspd : système sécant postérieur droit; S : ligne de suture; BrSq : branche squelettique; Sspg : système sécant postérieur gauche.

Ce cilié cylindroïde est arrondi à ses deux extrémités (115-203 × 96-160 μm). Il présente une légère constriction équatoriale (Figure 2). Très transparent, le cytoplasme présente deux rangées de quatre vacuoles contractiles chacune, situées de part et d’autre du macronoyau. Un micronoyau globuleux (1,5 à 4 μm de diamètre) flanque un macronoyau axial et rubané. Ce macronoyau est fréquemment très éloigné des extrémités de la cellule et mesure en moyenne 159,5 μm de longueur sur 5,2 μm de largeur (Figure 2A)

On dénombre 50 à 98 cinéties sur la face inférieure et 51 à 79 cinéties sur la face supérieure. Au pôle antérieur, on note une zone de suture des cinéties en forme d’accolade se prolongeant latéralement par deux systèmes sécants (Figure 2B). L’affrontement des stries ciliaires au pôle postérieur dessine une ligne de suture se prolongeant en fourche par deux systèmes sécants de chaque côté. Du côté droit, l’un des systèmes sécants remonte considérablement sur la face inférieure. Du côté gauche, la fourche formée par le prolongement des deux systèmes sécants s’observe nettement (Figure 2C).

Au pôle antérieur, et toujours légèrement déporté sur la face inférieure, on observe un appareil squelettique formé par un élément en V dont les branches inégales forment un angle presque plat. Il possède une architecture dissymétrique car, uniquement sur la branche gauche, s’insère une quinzaine de fibres squelettiques situées dans le prolongement des cinéties. Le sommet de l’élément en V porte une pointe légèrement recourbée (Figure 2B).

### *Paracoelophrya polymorphus* n. sp.

**Figure 3.**
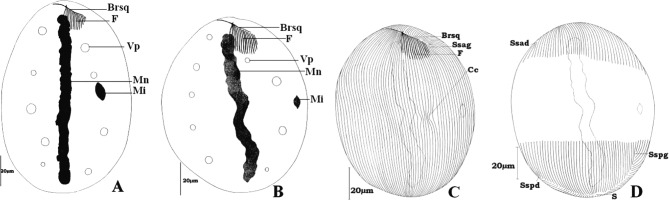
*Paracoelophrya polymorphus* n. sp. A : morphologie générale (forme ovoïde); B : morphologie générale (forme sphéroïde); C : ciliature de la face inférieure; D : ciliature de la face supérieure. Mn : macronoyau; Mi : micronoyau; Vp : vacuole pulsatile; F : fibres squelettiques; Cc : constriction des cinéties; S : ligne de suture; BrSq : branche squelettique; Sspg : système sécant postérieur gauche; Ssag : système sécant antérieur gauche; Ssad : système sécant antérieur droit; Sspd : système sécant postérieur droit.

C’est une cellule ovoïde (Figure 3A) ou sphéroïde de petite taille (Figure 3B), arrondie à ses deux pôles. Pendant l’intercinèse, elle mesure 95-125 × 70-102 μm. Elle présente un macronoyau axial et rubané et un micronoyau lenticulaire et médian de 3,0 μm de diamètre. L’appareil excréteur est formé par deux rangées de huit à 16 vacuoles disposées de part et d’autre du macronoyau. Deux formes se distinguent : la forme ovoïde (114,8 μm de long sur 108,1 μm de large) et la forme sphéroïde (111,4 μm de long sur 86 μm de large).

Le revêtement ciliaire est constitué par une quarantaine de cinéties bipolaires recouvrant uniformément chaque face (Figure 3C, D). Elles déterminent au pôle postérieur, une aire glabre très discrète prolongée par deux systèmes sécants, droit et gauche. Au pôle antérieur, on note une ligne de suture recourbée en arc dissymétrique. Cette zone de raccord se prolonge en deux systèmes sécants, l’un à droite et l’autre à gauche. L’appareil squelettique est formé par une branche squelettique arquée. Cette branche est toujours plus longue du côté du micronoyau et porte de ce côté une dizaine de fibres squelettiques formées par simple épaississement des cinétodesmes. Le cytosquelette est légèrement déporté du côté de la face inférieure (Figure 3A, B).

### *Paracoelophrya ebebdensis* n. sp.

**Figure 4.**
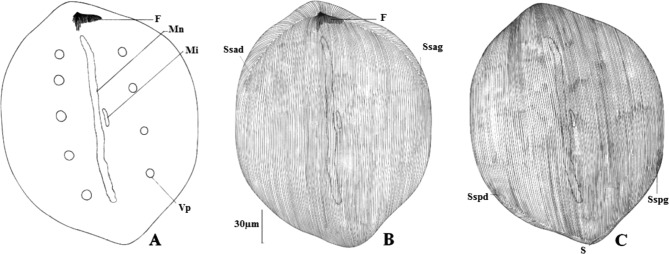
*Paracoelophrya ebebdensis* n. sp. A : morphologie générale; B: ciliature de la face inférieure; C : ciliature de la face supérieure. Mn : macronoyau; Mi : micronoyau; Vp : vacuole pulsatile; F : fibres squelettiques; S : ligne de suture; BrSq : branche squelettique; Sspg : système sécant postérieur gauche; Sspd : système sécant postérieur droit; Ssag : système sécant antérieur gauche; Ssad : système sécant antérieur droit.

De forme ovoïde, la cellule mesure en moyenne, 199 μm de long sur 159,5 μm de large pendant l’interphase (Figure 4). Elle a un macronoyau filiforme, axial et légèrement éloigné des pôles. Ce macronoyau porte, presque plaqué contre lui, dans la partie médiane, un micronoyau de faible diamètre (2,3 μm). Sept à 16 vacuoles contractiles se disposent en deux rangées de trois à huit vacuoles chacune de part et d’autre du macronoyau (Figure 4A).

Les cinéties sont très serrées, longitudinales et régulièrement espacées. On en compte 173 à 243 au total. Les raccords antérieur et postérieur des cinéties des deux faces se prolongent longuement en deux systèmes sécants chacun (Figure 4B, C).

Son appareil squelettique, axialement situé au pôle antérieur de la cellule, est constitué par un élément en V dont l’une des branches est pratiquement inexistante (Figure 4A, B). Il porte, uniquement du côté gauche, 15 à 25 fibres squelettiques situées dans le prolongement des cinéties. Les quatre à cinq fibres situées dans l’axe sont plus longues. Cette longueur diminue progressivement jusqu’à l’extrémité effilée de la branche squelettique.

#### • Discussion

##### *Paracoelophrya falcifera* n. sp.

L’appareil squelettique de l’espèce étudiée est typique du genre *Paracoelophrya*. Elle se rapproche de *P. almae* de Puytorac, 1969, par l’absence de système G et de cinétie A observée chez *P. intermedia* de Puytorac, 1969. Elle s’en éloigne par la constriction médiane de la cellule, par un plus grand nombre de cinéties, ce qui nous paraît justifier la création d’une nouvelle espèce.

##### *Paracoelophrya polymorphus* n. sp.

De par sa silhouette (ovoïde ou sphérique) et l’architecture de son armature squelettique possédant un petit crochet légèrement proéminent, placé sur une sorte d’élément en V dont les branches forment un angle presque plat, portant unilatéralement une dizaine de fibres squelettiques, cette espèce appartient typiquement au genre *Paracoelophrya* de Puytorac, 1969. Elle se différencie nettement des espèces *P. intermedia*, *P. almae* et *P. falcifera* par l’existence de deux systèmes sécants postérieurs au lieu de quatre. La taille et le nombre de cinéties sont plus petits que dans ces espèces. L’espèce est tenue pour nouvelle et nous la nommons *P. polymorphus*, du fait, non seulement de sa forme variable, mais aussi de sa spécificité aux *Alma* de petite taille récoltés à Nkolda et à Nkolbikogo.

##### *Paracoelophrya ebebdensis* n. sp.

Cette espèce diffère des autres espèces par une taille plus petite et un nombre de cinéties nettement plus grand. Elle est nommée ainsi du fait de sa présence à Ebebda

#### • Diagnose et localisation (Tableau I)

**Tableau I.** Comparaison des ciliés du genre *Paracoelophrya*.

	*P. intermedia*	*P. almae*	*P. falcifera* n. sp.	*P. polymorphus* n. sp.	*P. ebebdensis* n. sp.
Taille en	100 x 60	80 x 51	Constriction équatoriale 115–203 x 96–160	95–125 x 70–102	109 x 159
Vacuoles	2 rangées	1 rangée	2 rangées	2 rangées	2 rangées
Cytosquelette	Petit crochet + 15 fibres courtes	Cytosquelette à peine marqué	Petit cytosquelette + 18 fibres	En forme de V dissymétrique + 10 fibres	En forme de V très dissymétrique + 24 fibres
Cinéties	120–123	82–85	130–148	78–82	173–248
Systèmes sécants	2 systèmes sécants antérieurs longs	2 systèmes sécants antérieurs courts	2 systèmes sécants antérieurs longs	2 systèmes sécants courts antérieurs	2 systèmes sécants longs antérieurs
	4 systèmes sécants Postérieurs	4 systèmes sécants postérieurs	4 systèmes sécants postérieurs	2 systèmes sécants postérieurs	2 systèmes sécants postérieurs
Origine	Alma emint Gabon	Alma sp. Gabon	Alma nilotica Cameroun	Alma emini Cameroun	Alma emini Cameroun

##### *Paracoelophrya falcifera* n. sp.

Vit dans l’intestin d’*A. nilotica*. Cellule cylindroïde, arrondie à ses deux extrémités : 115-190 × 110-203 μm; deux rangées de huit à 16 vacuoles contractiles; 50-98 cinéties sur la face supérieure; 51-79 cinéties sur la face inférieure. Présente une légère torsion de sa moitié antérieure

Localisation : l’hôte est récolté sur les berges de la rivière Mia (affluent de la Mefou) à Nkolda, à environ 16 km au Sud-sud-ouest de Yaoundé. Caractéristiques écologiques du sol : pH 4,1; humidité 35,36%; matière organique 12,6%.

##### *Paracoelophrya polymorphus* n. sp.

Commensal du tube digestif d’*A. emini*. Cellule tantôt ovoïde tantôt sphéroïde : 95-125 × 70-102 μm; deux rangées de huit à 16 vacuoles pulsatiles; 39-55 cinéties sur la face supérieure; 38-49 cinéties sur la face inférieure; 11-16 fibres squelettiques. Colonise toute la région antérieure et moyenne de 86,66% des *Alma* de petite taille récoltés à Nkolbikogo avec une abondance de 62 ciliés par ver.

Localisation : l’hôte est récolté sur les berges de la rivière Mia (affluent de la Mefou) à Nkolda, à environ 16 km au Sud-sud-ouest de Yaoundé. Caractéristiques écologiques du sol : pH 4,1; humidité 35,36%; matière organique 12,6%.

##### *Paracoelophrya ebebdensis* n. sp.

Vit dans l’intestin d’*A. emini*. Cellule ovoïde : 145-225 × 125-190 μm; deux rangées de sept à 16 vacuoles pulsatiles; 89-123 cinéties sur la face supérieure; 87-122 cinéties sur la face inférieure; 15-22 fibres squelettiques.

Fréquent et abondant chez les vers de petite taille (ils sont retrouvés dans 96,66% de vers, avec une abondance de 283 ciliés par ver). Par contre, 26,6% de vers de grande taille en sont porteurs, avec une abondance de 24 ciliés par ver.

Localisation : l’hôte est récolté sur les berges du fleuve Sanaga à Ebebda, à 60 km au nord de Yaoundé. Caractéristiques écologiques du sol : pH 4,9; humidité 33,6%; matière organique 2,7%.

### Description des espèces du genre *Dicoelophrya*

Nous présentons trois espèces du genre *Dicoelophrya* dont deux nouvelles : *D. nkoldensis* et *D. mediovacuolata*.

#### *Dicoelophrya almae* de Puytorac et Dragesco, 1969

De localisation très précise dans le tube digestif des Oligochètes Glossoscolecidae du genre *Alma* récolté à Ebebda, Nkolbikogo et Nkolda, la fréquence est de l’ordre de 100% avec une abondance de 72 ciliés par ver à Ebebda. Ces valeurs sont respectivement 86,66% et 147 ciliés par ver à Nkolbikogo et Nkolda.

**Figure 5.**
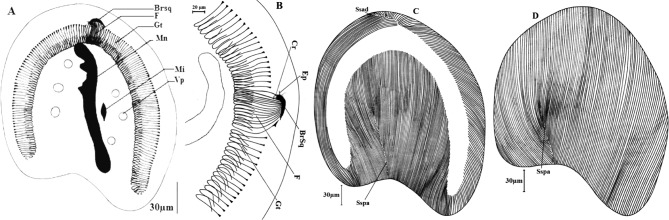
*Dicoelophrya almae* de Puytorac et Dragesco, 1969 A : morphologie générale; B : détail du pôle antérieur; C : ciliature de la face inférieure; D : ciliature de la face supérieure. Mn : macronoyau; Mi : micronoyau; Vp : vacuole pulsatile; F : fibres squelettiques; Gt : gouttière; Cr : crochet; BrSq : branche squelettique; Ep: épine; Sspa : système sécant postérieur axial; Ssad : système sécant antérieur droit.

**Figure 6.**
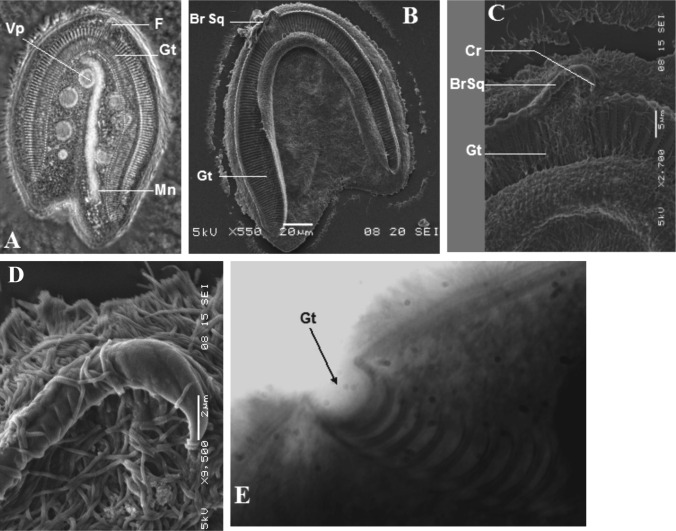
Photographies de *Dicoelophrya almae* de Puytorac et Dragesco, 1969. A : morphologie générale; image de microscopie à contraste de phase, superposée à la fluorescence de l’appareil nucléaire coloré au DAPI; B-E : microscopie électronique à balayage : B : topographie générale de la gouttière; C : détail du pôle antérieure; D : mise en évidence de la branche squelettique; E : détail du pôle postérieur gauche (vu par le pôle antéro-inférieur) montrant une topographie de la gouttière (observation vitale en contraste de phase). Mn : macronoyau; Vp : vacuole pulsatile; F : fibres squelettiques; Gt : gouttière; Cr : crochet; BrSq : branche squelettique.

Ce cilié a une forme générale en cloche, arrondie au pôle antérieur et présentant un pôle postérieur fourchu. Il est légèrement aplati sur sa face inférieure, et montre une convexité nette sur sa face supérieure. Sa face inférieure porte une dépression en forme de fer à cheval très caractéristique, marquant la position d’un appareil squelettique formé par les arceaux d’une succession de fibres l’ensemble donnant l’allure d’une gouttière surmontée par une branche squelettique terminée en un crochet. La cellule mesure en moyenne 165 μm de long sur 137 μm de large. Le pôle antérieur est arrondi. Le pôle postérieur présente une fourche de longueur inégale et plus ou moins ouverte. La fourche du côté gauche est plus longue (Figures 5A, 6A). Le macronoyau est légèrement oblique et excentré. La moitié antérieure porte des digitations plus ou moins marquées (Figures 5A, 6A). Il est flanqué dans sa zone équatoriale par un micronoyau lenticulaire de 4 μm de diamètre.

Sur la face inférieure, en moyenne 160 cinéties sont légèrement radiaires et s’affrontent dans la région axiale en un système sécant postérieur. Les cinéties du côté gauche et celles du côté droit s’affrontent au pôle antérieur en un court système sécant très caractéristique (Figure 5C). Sur la face supérieure, les cinéties recouvrent la cellule de manière homogène et s’affrontent postérieurement en un système sécant excentré du côté gauche (Figure 5D).

L’appareil squelettique est constitué par 160 fibres squelettiques dont les arceaux sont recourbés de manière non symétrique à leurs extrémités, formant ainsi deux lèvres recouvrant les bords d’une dépression qui constitue la gouttière (Figure 6E). Le diamètre de cette gouttière diminue progressivement vers son extrémité postérieure (Figures 5A, 6A, B). La gouttière en fer à cheval est surmontée dans sa partie axiale par une hampe sur laquelle s’appuient une quinzaine de fibres squelettiques, dont les quatre premiers du côté droit sont particulièrement robustes (Figures 5B, 6C). Cette hampe en faucille est courte et plus effilée du côté gauche. Elle se replie légèrement à son extrémité droite en un crochet puissant (Figures 5B, 6C, D).

#### *Dicoelophrya nkoldensis* n. sp.

Ce cilié vit dans toute la région antérieure des *Alma* de petite taille, récoltés à Nkolbikogo et à Nkolda. Sa fréquence est de 96,66% avec une abondance de 132 ciliés par ver. C’est un cilié arrondi au pôle antérieur et tronqué au pôle postérieur. La face supérieure est nettement convexe. La face inférieure est légèrement aplatie et porte une gouttière adhésive repliée en forme de fer à cheval, marquée par une dépression. Une vingtaine de vacuoles s’éparpillent dans le cytoplasme et sans ordre précis de part et d’autre du macronoyau.

**Figure 7.**
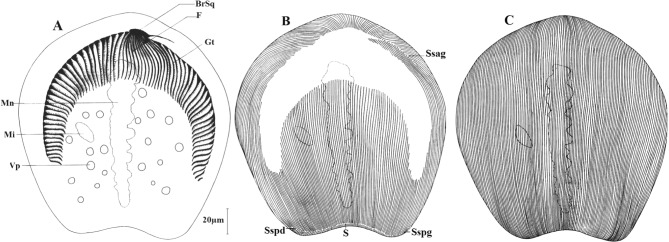
*Dicoelophrya nkoldensis* n. sp. A : morphologie générale; B : ciliature de la face inférieure; C : ciliature de la face supérieure. Mn : macronoyau; Mi : micronoyau; Vp : vacuole pulsatile; F : fibres squelettiques; Gt : gouttière; BrSq : branche squelettique; Sspg : système sécant postérieur gauche; Sspd : système sécant postérieur droit; Ssag : système sécant antérieur gauche; S : ligne de suture.

**Figure 8.**
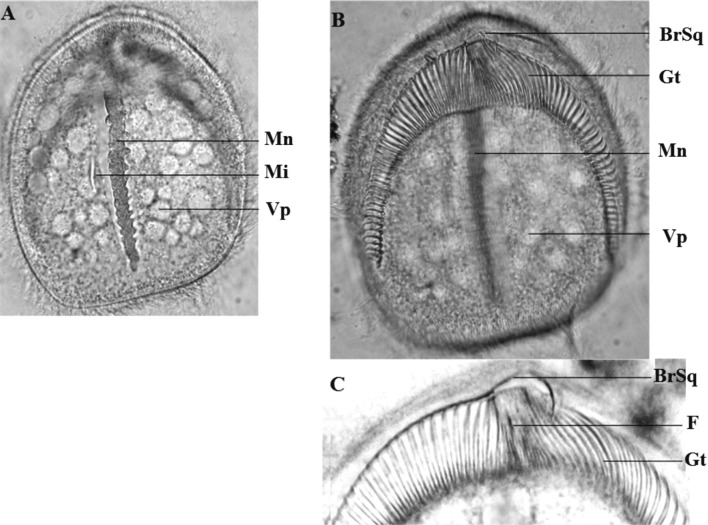
Photographies de *Dicoelophrya nkoldensis* n. sp. A : morphologie générale mettant en évidences la disposition des vacuoles; B : mise en évidence de la gouttière; C : détail du pôle antérieur montrant la branche squelettique. Images prises au microscope optique à contraste de phase. Mn : macronoyau; Mi : micronoyau; Vp : vacuole pulsatile; F : fibres squelettiques; Gt : gouttière; BrSq : branche squelettique.

Ce dernier est axial et légèrement éloigné des pôles. Il mesure en moyenne 108,4 μm de long pour une largeur de 13 μm. Le macronoyau porte des digitations plus ou moins marquées. Il est flanqué d’un micronoyau lenticulaire ou globuleux de 4 μm de diamètre et situé dans la zone équatoriale de la cellule (Figures 7A, 8A). La cellule est douée d’une grande mobilité et d’une grande faculté d’adhérence au substrat.

La ligne de suture antérieure est déportée sur la face inférieure et se prolonge du côté gauche par un long système sécant, du côté droit; cette ligne de suture coïncide avec l’emplacement de la gouttière dont elle limite le bord antérieur. Entre les branches de la gouttière on compte environ 75 cinéties limitant au pôle postérieur une ligne de suture qui se prolonge en deux systèmes sécants courts (Figure 7B). La face supérieure de la cellule est recouverte de manière homogène par près de 122 cinéties méridiennes et bipolaires (Figure 7C).

Le cytosquelette est constitué par une hampe massive repliée en son extrémité gauche en un crochet (Figure 7C). Sur cette hampe s’appuie une quinzaine de fibres squelettiques qui se prolongent dans une dépression sous-jacente. C’est cette concavité qui s’étend latéralement en deux “rigoles” dont les fibres repliées en arceaux forment une gouttière presque symétrique, la branche droite étant légèrement plus longue. On compte au total 71 fibres squelettiques dans cette gouttière (Figures 7A, 8B, C).

#### *Dicoelophrya mediovacuolata* n. sp.

**Figure 9.**
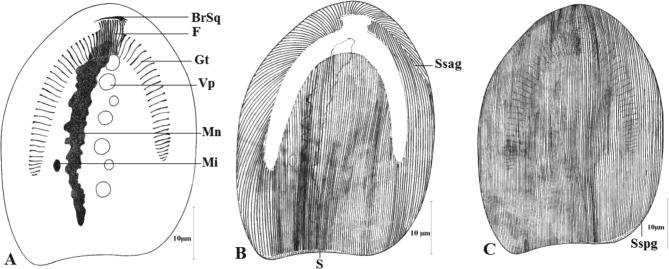
*Dicoelophrya mediovacuolata* n. sp. A : morphologie générale; B : ciliature de la face inférieure; C : ciliature de la face supérieure. Mn : macronoyau; Mi : micronoyau; Vp : vacuole pulsatile; F : fibres squelettiques; Gt : gouttière; BrSq : branche squelettique; Sspg : système sécant postérieur gauche; Ssag : système sécant antérieur gauche; S : ligne de suture.

L’hôte est un *Alma* de petite taille récolté sur les berges du fleuve Sanaga à Ebebda, à 60 km au nord de Yaoundé (Tableau II). On note une forte fréquence d’infestation (93,33% des vers étudiés en sont por- teurs), mais une faible abondance. C’est un cilié de très petite taille (37 à 59 μm de long sur 30 à 50 μm de large) dont le pôle antérieur est arrondi tandis que le pôle postérieur est légèrement tronqué à l’oblique. Le macronoyau est en forme de massue, portant des digitations plus ou moins marquées dans sa partie antérieure. Il est flanqué à son tiers postérieur par un micronoyau globuleux qui lui est presque accolé. Cet appareil nucléaire est excentré par rapport à l’axe de symétrie bilatérale de la cellule. On dénombre deux à neufs vacuoles pulsatiles disposées en une rangée longitudinale dans l’axe du cilié (Figure 9A).

**Tableau II.** Comparaison des ciliés du genre *Dicoelophrya*.

	*D. calliste*	*D. almae*	*D. nkoldensis* n. sp.	*D. mediovacuolata* n. sp.
Taille en µm	85–120 x 45–60	120–180 x 90–100	132–220 x 90–210	37–59 x 30–50
Vacuoles	2 rangées	2 rangées	Disséminées	1 rangée axiale
Cytosquelette	Hampe 10 µm + 12 fibres	Hampe 10 µm + 12 fibres	Hampe + 13 fibres	Hampe + 11–15 fibres
Cinéties	170–180	280 en moyenne	200 en moyenne	168 en moyenne
Gouttière	Gouttière dissymétrique 20–22 arceaux	Gouttière dissymétrique 142–160 arceaux	Gouttière presque symétrique 71 arceaux	Gouttière dissymétrique 64 arceaux
Origine	*Alma emini* Gabon	*Alma* sp. Gabon et Cameroun	*Alma* sp. Cameroun	*Alma* sp. Cameroun

**Figure 10.**
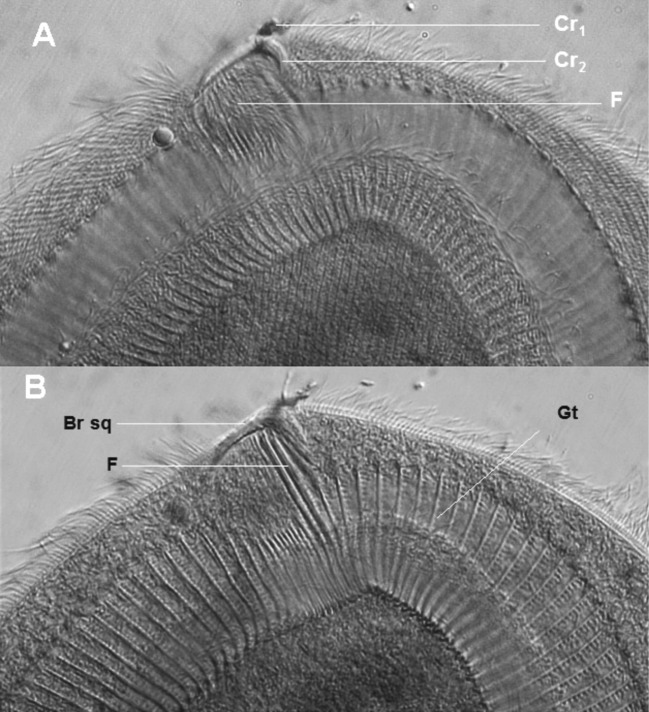
Photographie de *Dicoelophrya mediovacuolata* n. sp. A et B : détail du pôle montrant l’architecture de l’appareil squelettique (observation en microscopie confocale). Gt : gouttière; BrSq : branche squelettique; F : fibres squelettiques; Cr1, Cr2 : crochets.

La topographie ciliaire est faite de 88 cinéties méridiennes recouvrant la face supérieure de manière homogène. Les extrémités de ces cinéties se déportent antérieurement sur la face inférieure, délimitant une aire glabre occupée par le cytosquelette (Figure 9B, C). La ligne de suture antérieure se prolonge longuement du coté gauche par un système sécant bien marqué. La face inférieure porte, entre les branches de la gouttière, 64 cinéties en moyenne. Les cinéties des deux faces s’affrontent postérieurement en une longue ligne de suture qui se prolonge latéralement par deux systèmes sécants très discrets (Figure 9B, C). Le cytosquelette est constitué par une branche squelettique robuste en faucille avec un crochet dont l’extrémité droite est effilée. Cette hampe est soutenue par 11 à 15 fibres squelettiques. D’autres fibres, repliées en arceaux s’étendent latéralement, formant les armatures d’une gouttière bilatérale, non symétrique, se prolongeant plus longuement du coté droit, proche du micronoyau. Le cytosquelette est constitué en moyenne de 64 fibres squelettiques (Figure 10A, B).

#### • Discussion

*Dicoelophrya almae* de Puytorac et Dragesco, 1969 L’organisation du cytosquelette est caractéristique des ciliés du genre *Dicoelophrya* de Puytorac & Dragesco, 1969. La morphologie générale rappelle celle *D. almae* de Puytorac & Dragesco, 1969; bien que le cilié ici décrit accuse quelques particularités, la taille est plus grande chez nos spécimens (130-215 × 96-190 μm contre 120-180 × 90-100 μm), et la présence sur chaque face un système sécant postérieur, proche de l’axe de symétrie bilatérale de la cellule. Par sa morphologie générale il peut être confondu à première vue avec *D. mediovacuolata* n. sp. Le pôle antérieur est arrondi, l’appareil nucléaire excentré et la gouttière est en forme de fer à cheval; il s’en démarque cependant par sa gouttière plus longue et la forme en fourche de son pôle postérieur. En dépit de quelques variations observées, nous pensons qu’il est judicieux de maintenir pour ce cilié, sa dénomination originale : *D. almae* de Puytorac & Dragesco, 1969.

##### *Dicoelophrya nkoldensis* n. sp.

Ce cilié diffère de *D. calliste* de Puytorac et Dragesco, non seulement par sa taille plus grande (132-220 × 90-210 μm, alors qu’elle est de 85-120 × 45-60 μm), mais aussi par le système vacuolaire (vacuoles disséminées au lieu de deux rangées), et par un plus grand nombre d’arceaux squelettiques. Nous pensons qu’il s’agit d’une nouvelle espèce : *D. nkoldensis* spécifique des *A. nilotica* récoltés à Nkolda.

##### *Dicoelophrya mediovacuolata* n. sp.

Ce cilié est bâti sur le même plan d’organisation que *D. calliste*. Son pôle antérieur est arrondi et son pôle postérieur tronqué. Les fibres squelettiques forment une gouttière en fer à cheval. La topographie ciliaire est analogue. Il n’en diffère que par sa taille beaucoup plus réduite (37-59 × 30-50 μm contre 168,2 × 132,7 μm), par l’organisation de l’appareil excréteur constitué, contrairement aux autres espèces, d’une seule rangée de deux à neuf vacuoles pulsatiles alignées dans la partie axiale de la cellule, et par la disposition de l’appareil nucléaire, plutôt excentré. Cette organisation de l’appareil nucléaire rapproche cette dernière espèce de *D. almae*, avec laquelle elle pourrait être confondue si ce n’était sa taille très réduite, l’organisation de son appareil vacuolaire (une rangée axiale au lieu de deux) et sa branche squelettique à deux crochets. C’est en raison de ces différences que nous avons jugé nécessaire de créer pour ce cilié, une nouvelle espèce que nous dénommons : *D. mediovacuolata*. Par ailleurs, notons que cette espèce n’a été retrouvée que chez *A. nilotica* récoltés à Ebebda. De plus, la connaissance précise de la topographie de l’armature squelettique dans les genres *Paracoelophrya*, *Metaracoelophrya*, *Coelophrya* et *Dicoelophrya* témoigne de manière indéniable de l’homogénéité de la sous-famille des Metaracoelophryinae. Ces observations soulignent la possibilité d’une relation phylétique entre ces genres.

#### • Diagnose et localisation (Tableau II)

##### *Dicoelophrya mediovacuolata* n. sp.

Commensal du tube digestif d’*Alma* sp. (37-59 × 30- 50 μm). Une vingtaine de vacuoles pulsatiles dispersées dans le cytoplasme et sans ordre apparent; 72- 103 cinéties sur la face supérieure; 53-79 cinéties sur la face inférieure; 53-79 fibres squelettiques forment la gouttière; 11 à 15 des fibres de la gouttière s’appuient axialement sur la branche squelettique antérieure. On note une forte fréquence d’infestation (93,33% des vers étudiés en sont porteurs), mais une faible abondance. Localisation : l’hôte est récolté sur les berges du fleuve Sanaga à Ebebda, à 60 km au nord de Yaoundé. Caractéristiques du sol : pH 4,9; humidité 33,6%; matière organique 2,7%.

##### *Dicoelophrya nkoldensis* n. sp

Vit dans l’intestin d’*Alma* sp. Cellule ovoïde : 132-220 × 90-210 μm; une vingtaine de vacuoles pulsatiles dispersées dans le cytoplasme et sans ordre apparent; 108-136 cinéties sur la face supérieure; 67-74 cinéties sur la face inférieure; 67-74 fibres squelettiques. Spécifique des vers de petite taille de Nkolda. Fréquence : 96,66%; abondance : 132 ciliés par ver.

Localisation : l’hôte est récolté sur les berges de la rivière Mia (affluent de la Mefou) à Nkolda, à environ 16 km au Sud-sud-ouest de Yaoundé. Caractéristiques du sol : pH 4,1; humidité 35,36%; matière organique 12,6%.

## Discussion Générale

**Figure 11.**
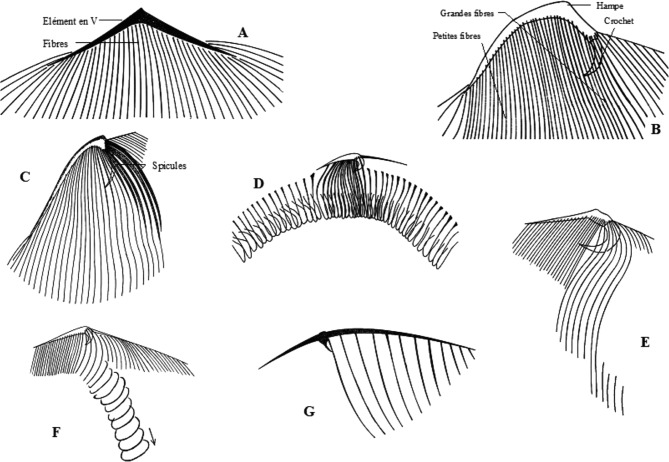
Cytosquelette des genres représentatifs de la famille des Radiophryida (d’après de Puytorac et Dragesco, 1969, sauf D). A : *Radiophrya;* B : *Metaradiophrya;* C : *Cheissinophrya;* D : *Dicoelophrya;* E : *Metaracoelophrya;* F : *Coelophrya;* G : *Paracoelophrya*.

Les quatre genres connus de Metaracoelophryinae, tous parasites des Glossoscolecidae, forment un ensemble homogène dans lequel de Puytorac 1972 notait une régression de l’ensemble du cytosquelette chez les *Paracoelophrya*. La figure 11 permet en effet une comparaison du squelette de quelques genres de Radiophryidae de Puytorac, 1972 : *Radiophrya* Rossolimo, 1926; *Metaradiophrya* Hedenreich, 1935; *Cheissinophrya* de Puytorac, 1969; *Dicoelophrya* de Puytorac & Dragesco, 1969; *Coelophrya* de Puytorac & Dragesco, 1969 et *Paracoelophrya* de Puytorac, 1969, dont nous rappelons les caractères. Dans tous les genres, la cellule est pourvue à la partie antérieure de ce que de Puytorac a nommé un élément en V, d’où partent, sur la face inférieure, des fibres squelettiques ecto-endoplasmiques longeant les cinéties.

Chez *Radiophrya* (Figure 11A) les deux branches de l’élément en V sont symétriques et les fibres s’étendent sur la plus grande partie de la face inférieure.

Chez *Metaradiophrya* (Figure 11B) la branche droite de V est une hampe robuste terminée par un crochet massif et donne insertion à une série de fibres. La branche gauche résulte de la juxtaposition des extrémités antérieures des fibres puissantes dans la région médiane de la cellule et diminuent de diamètre vers la gauche. L’ensemble des fibres s’étend sur une grande partie de la face inférieure.

Chez *Cheissinophrya* (Figure 11C), on retrouve la hampe droite très puissante avec un crochet marqué. Depuis la base de ce dernier et sur la gauche, un filin de petit diamètre, à peu près symétrique de la hampe, est suivi vers la gauche de fibres dont le diamètre va en diminuant. La partie gauche de l’élément en V réduite ne porte que de courtes fibres.

Chez *Dicoelophrya* (Figure 11D), la hampe courte, pourvue d’un crochet porte une série de fibres médianes. Sur les côtés droit et gauche, les fibres s’épaississent localement en un arceau et leur succession forme une gouttière. Les gouttières droite et gauche se réunissent en un ensemble en fer à cheval. Figure 11.

Chez *Metaracoelophrya* (Figure 11E), la partie droite de l’élément en V est peu marquée, mais il y a un crochet massif. Sur la droite de l’élément en V sont insérées des fibres en rideaux de plus en plus courts vers la droite et correspondant à l’ensemble d’une aire thigmotactique. À la base du crochet et vers la gauche (où la partie gauche de l’élément en V est une ligne fine), s’insèrent quelques fibres puissantes tordues à mi-parcours en forme d’arceaux, l’ensemble amorçant une gouttière obliquement dirigée vers la partie postérieure de la cellule, mais très loin de l’atteindre.

Chez *Coelophrya* (Figure 11F), la forme du cytosquelette est comparable mais la gouttière s’étend jusqu’à la partie postérieure où elle tend à devenir un canal quasi clos, puis une simple dépression.

Chez *Paracoelophrya* (Figure 11G), l’élément en V est très aplati avec une simple pointe à la jonction d’une partie droite aux fibres non détectables et d’une partie gauche porteuse de quelques courtes fibres de plus en plus fines vers l’extrémité gauche.

Selon de Puytorac, tous les Hoplitophryida forment un groupe homogène avec deux tendances évolutives du cytosquelette en V : soit simplification et réduction pouvant aboutir à la disparition, soit complication et développement du système. de Puytorac (1954) considère les Hoplitophryida comme des Oligohymenophora proches des Scuticociliés et Thigmotriches. Des études phylogénétiques en cours basées sur les ADNr pourront préciser si cette hypothèse est vérifiée, et si les Hoplitophryida sont proches des Anoplophryida, astomes dépourvus de cytosquelette.

## References

[R1] Blanc J. Étude cytophotométrique des périodes de duplication de l’ADN dans l’appareil nucléaire de *Paramecium caudatum*. Protistologica, 1965, 1, 11–15.

[R2] Cheissin E. Morphologische und systematische studien uber Astomata aus dem Baikalsee. Archiv Protistenk, 1930, 70, 531–618.

[R3] de Puytorac P. Contribution à l’étude cytologique et taxonomique des Infusoires Astomes. Annales des Sciences Naturelles, Zoologie, 1954, 11, 85–270.

[R4] de Puytorac P. Les ciliés astomes Hoplitophryidae I. Description de nouvelles espèces. Protistologica, 1970, 5, 255–268.

[R5] de Puytorac P. Les ciliés astomes Hoplitophryidae. Révision de la systématique de ce groupe. Protistologica, 1972, 8, 5–42.

[R6] de Puytorac P. Proposition d’une classification du phylum ciliophora. Doflein, Réunion de systématique, Clermont-Ferrand 1901 (Centre de Recherche Académique de Paris). Centre de Recherche Académique de Paris, 1974, 278, 5–27.

[R7] de Puytorac P. Sous-classe des Astomatia Schewiakoff, 1896, *in*: Traité de Zoologie, Infusoires Ciliés, vol 2 P. de Puytorac (Ed.), Masson, Paris, 1994, 751–787.

[R8] de Puytorac P. & Dragesco J. Quatre espèces nouvelles de ciliés astomes chez les *Alma emini* (Mchlsn) (ver Criodrilinae) du Gabon. Annales de la Station Biologique de Besse-en-Chandesse, 1968, 3, 259–266.

[R9] de Puytorac P. & Dragesco J. Description de six genres nouveaux de ciliés astomes Hoplitophryidae endoparasites de vers Glossoscolecidae au Gabon. Biologia Gabonica, 1969, 5, 5–27.

[R10] de Puytorac P. & Schrével J. Nouvelle espèce de ciliés Astome endoparasite d’Annélides Polychètes. Ann Fac Sc Clermont-Fd, 1965, 26, 85–99.

[R11] Fernandez Galiano D. Silver impregnation of ciliated protozoa: procedure yielding good results with the pyridinated silver carbonate method. Trans Am Microsc Soc, 1976, 95, 557–560. 65821

[R12] Fernandez Galiano D. The ammoniacal silver carbonate method as a general procedure in the study of protozoa from sewage (and other) waters. Wat Res, 1994, 28 (2), 495–496.

[R13] Fokam Z., Ngassam P., Boutin C.L. & Zébazé Togouet S.H. Trois espèces nouvelles de *Coelophrya*, Ciliés Astomes endocommensaux d’*Alma nilotica* (Oligochète terricole) du Cameroun. Bulletin de la Société d’Histoire Naturelle de Toulouse, 2008, 144, 27–33.

[R14] Ngassam P. Trois espèces nouvelles de ciliés Astomes des genres : *Almophrya* de Puytorac et Dragesco, 1968, *Maupasella* Cépède, 1910, *Njinella* nov. genre, endocommensaux d’Annélides oligochètes de la région de Yaoundé. Protistologica, 1983, 19 (1), 131–135.

[R15] Omodeo P. Oligochètes, *in*: La réserve naturelle intégrale du Mont Nimba. Mémoires de l’Institut Français d’Afrique Noire, Douala, 1958, 53, 9–109.

[R16] Rossolimo L. Parasitische Infusorien aus dem Baikalsee. Arch F Protistenk, 1926, 54, 468–510.

[R17] Williamson D.H. & Fennell D.J. The use of fluorescent DNA-binding agent for detecting and separating yeast mitochondrial DNA, *in*: Methods in Cell Biology, vol 12 D.M. Prescott (ed.), Academic Press, New York, 1975, 335–351. 1105070 10.1016/s0091-679x(08)60963-2

